# Molecular characteristics and zoonotic potential of enteric protists in domestic dogs and cats in Egypt

**DOI:** 10.3389/fvets.2023.1229151

**Published:** 2023-07-06

**Authors:** Ehab Kotb Elmahallawy, Ahmed Gareh, Akram Abu-Okail, Pamela C. Köster, Alejandro Dashti, Jamal Asseri, Asmaa Aboelabbas Gouda, Murad A. Mubaraki, Sara Abdel-Aal Mohamed, Yasser M. Mohamed, Ehssan Ahmed Hassan, Mohamed Elgendy, Carolina Hernández-Castro, Begoña Bailo, David González-Barrio, Lihua Xiao, David Carmena

**Affiliations:** ^1^Departamento de Sanidad Animal, Grupo de Investigación en Sanidad Animal y Zoonosis (GISAZ), Facultad de Veterinaria, Universidad de Córdoba, Córdoba, Spain; ^2^Department of Zoonoses, Faculty of Veterinary Medicine, Sohag University, Sohag, Egypt; ^3^Department of Parasitology, Faculty of Veterinary Medicine, Aswan University, Aswan, Egypt; ^4^Department of Veterinary Medicine, College of Agriculture and Veterinary Medicine, Qassim University, Buraydah, Saudi Arabia; ^5^Parasitology Reference and Research Laboratory, National Centre for Microbiology, Majadahonda, Spain; ^6^Department of Biology, College of Science and Humanities, Shaqra University, Dawadmi, Saudi Arabia; ^7^Department of Parasitology, Faculty of Veterinary Medicine, Zagazig University, Zagazig, Egypt; ^8^Clinical Laboratory Sciences Department, College of Applied Medical Sciences, King Saud University, Riyadh, Saudi Arabia; ^9^Department of Parasitology, Faculty of Veterinary Medicine, Assiut University, Assiut, Egypt; ^10^Department of Parasitology, Faculty of Medicine, Assiut University, Assiut, Egypt; ^11^Department of Biology, College of Science and Humanities in Al-kharj, Prince Sattam Bin Abdulaziz University, Alkharj, Saudi Arabia; ^12^Department of Zoology, Faculty of Science, Suez Canal University, El-Sheikh Zayed, Ismailia, Egypt; ^13^Department of Surgery, Anesthesiology and Radiology, Faculty of Veterinary Medicine, Kafrelsheikh University, Kafrelsheikh, Egypt; ^14^Parasitology Group, Faculty of Medicine, Academic Corporation for the Study of Tropical Pathologies, University of Antioquia, Medellín, Colombia; ^15^College of Veterinary Medicine, South China Agricultural University, Guangzhou, China; ^16^Center for Biomedical Research in Infectious Diseases, Carlos III Health Institute (ISCIII), Madrid, Spain

**Keywords:** enteric parasites, epidemiology, zoonoses, genotyping, small subunit ribosomal RNA gene, 60 kDa glycoprotein

## Abstract

**Introduction:**

Domestic dogs and cats can be a source of human infection by a wide diversity of zoonotic pathogens including parasites. Genotyping and subtyping tools are useful in assessing the true public health relevance of canine and feline infections by these pathogens. This study investigated the occurrence, genetic diversity, and zoonotic potential of common diarrhea-causing enteric protist parasites in household dogs and cats in Egypt, a country where this information is particularly scarce.

**Methods:**

In this prospective, cross-sectional study a total of 352 individual fecal samples were collected from dogs (*n* = 218) and cats (*n* = 134) in three Egyptian governorates (Dakahlia, Gharbeya, and Giza) during July–December 2021. Detection and identification of *Cryptosporidium* spp., *Giardia duodenalis*, *Enterocytozoon bieneusi*, and *Blastocystis* sp. were carried out by PCR and Sanger sequencing. Basic epidemiological variables (geographical origin, sex, age, and breed) were examined for association with occurrence of infection by enteric protists.

**Results and discussion:**

The overall prevalence rates of *Cryptosporidium* spp. and *G. duodenalis* were 1.8% (95% CI: 0.5–4.6) and 38.5% (95% CI: 32.0–45.3), respectively, in dogs, and 6.0% (95% CI: 2.6–11.4) and 32.1% (95% CI: 24.3–40.7), respectively, in cats. All canine and feline fecal samples analyzed tested negative for *E. bieneusi* and *Blastocystis* sp. Dogs from Giza governorate and cats from Dakahlia governorate were at higher risk of infection by *Cryptosporidium* spp. (*p* = 0.0006) and *G. duodenalis* (*p* = 0.00001), respectively. Sequence analyses identified host-adapted *Cryptosporidium canis* (*n* = 4, one of them belonging to novel subtype XXe2) and *G. duodenalis* assemblages C (*n* = 1) and D (*n* = 3) in dogs. In cats the zoonotic *C. parvum* (*n* = 5) was more prevalent than host-adapted *C. felis* (*n* = 1). Household dogs had a limited (but not negligible) role as source of human giardiasis and cryptosporidiosis, but the unexpected high frequency of zoonotic *C. parvum* in domestic cats might be a public health concern. This is the first molecular-based description of *Cryptosporidium* spp. infections in cats in the African continent to date. Molecular epidemiological data provided here can assist health authorities and policy makers in designing and implementing effective campaigns to minimize the transmission of enteric protists in Egypt.

## 1. Introduction

*Cryptosporidium* spp., *Giardia duodenalis*, *Enterocytozoon bieneusi*, and *Blastocystis* sp. are common zoonotic protists able to cause diarrhea and other gastrointestinal disorders in a wide range of animal species including humans (1–3). Human infection outcomes vary largely from asymptomatic to severe manifestations and even death. The most frequent clinical signs are abdominal discomfort, anorexia, acute and chronic diarrhea, nausea, and weight loss. Fever, vomiting, and bloody stool are less common (4–6). Extraintestinal manifestations including urticaria and other allergic diseases have also been reported for some of them (7). All four pathogens are fecal-orally transmitted after accidental ingestion of their transmissive stages (cysts, oocysts, spores) directly through contact with infected humans or animals or indirectly via consumption of contaminated water or fresh produce (8, 9).

*Cryptosporidium* spp., *G. duodenalis*, *Blastocystis* sp., and *E. bieneusi* display a large intra-species genetic diversity with marked differences in host specificity, range, zoonotic potential and even pathogenicity (10–12). Dogs and cats are commonly infected with *Cryptosporidium* spp. and *G. duodenalis* (13, 14), being primarily infected by host-adapted species/genetic variants including *Cryptosporidium canis* and *Cryptosporidium felis* and *G. duodenalis* assemblages C, D, and F. Despite the risk of zoonotic transmission of *Cryptosporidium* spp. and *G. duodenalis* from domestic dogs and cats is typically regarded as low (15–17), the sporadic but constant reporting of human infections caused by canine- and feline-adapted species/genotypes of these pathogens suggest that the role of dogs and cats as sources of human cryptosporidiosis and giardiasis should not be overlooked (18, 19).

The stramenopile *Blastocystis* sp. is a highly polymorphic protozoal parasite of uncertain pathogenicity commonly detected in fecal samples of humans and several other animal species. The parasite encompasses at least 36 subtypes (ST; ST1-ST17, ST21, ST23-ST40) (11, 20, 21). *Blastocystis* sp. is typically reported at relatively low (7%–9%) carriage rates in dogs and cats globally (22). Both host species have been shown to carry zoonotic STs including ST1–8, ST10, and ST14 (22), although the occurrence of zoonotic transmission events seems rare (23). Furthermore, *E. bieneusi* is an obligate intracellular fungus-like parasite with high genetic diversity among mammalian and avian hosts (24). Nearly 600 genotypes have been described within *E. bieneusi* (12), of which zoonotic genotypes A, BEB6, D, and TypeIV have been found circulating in domestic dogs and cats (25).

Domestic dogs and cats can carry a large variety of bacterial, viral, and parasitic (including protist) pathogens which can be transmitted to humans through bites, scratches, saliva, urine, feces, or contaminated surfaces. Therefore, understanding the frequency and molecular diversity of these pathogens is important to assess their zoonotic potential and public health relevance. In Egypt, information on the epidemiology of intestinal protist species of public and veterinary health relevance in canine and feline populations is scarce. Most of the studies conducted to date were based on conventional microscopy as screening method, and only few assessed the frequency and diversity of species/genotypes at the molecular level (Table 1) (26–39). It is therefore essential to conduct periodical surveys to provide updated information on the current status of these pathogens in domestic animals, which might be helpful to reduce the risk of potential zoonotic transmission events to humans. Under this approach, this molecular study investigated the occurrence, genetic diversity, and zoonotic potential of *Cryptosporidium* spp., *G. duodenalis*, *Blastocystis* sp., and *E. bieneusi* infection in domestic dogs and cats in three geographical areas of Egypt.

**Table 1:** Frequency and genetic diversity of *Cryptosporidium* spp., *Giardia duodenalis*, *Enterocytozoon bieneusi*, and *Blastocystis* sp. infections reported in canine and feline populations in Egypt, 1995–2022.

Pathogen	Host	Sample size (*n*)	Detection method	Prevalence (%)	Species (*n*)	Genotype (*n*)	References
*Cryptosporidium* spp.	Dog	50	CM	34.0	–	–	(26)
		50	PCR	24.0	*C. parvum* (5)	ND	
	Dog	395	CM	10.1	–	–	(27)
	Dog	130	CM	5.4	–	–	(28)
	Dog	60	CM	1.7	–	–	(29)
	Dog	20	CM, PCR	50.0	*C. parvum* (2)	ND	(30)
	Dog	27	CM	18.5	–	–	(31)
	Dog	27	CM	11.1	–	–	(32)
	Dog	25	CM	12.0	–	–	(33)
	Dog	685	CM	3.8	–	–	(34)
*Giardia duodenalis*	Dog	986	CM, PCR	8.5	–	D (4)	(35)
	Dog	395	CM	0.5	–	–	(27)
	Dog^a^	120	CM	1.7	–	–	(29)
	Dog^b^	60		31.7	–	–	
	Dog	685	CM	8.3	–	–	(34)
	Dog	27	CM	14.8	–	–	(31)
	Cat	113	CM	2.0	–	–	(36)
*Enterocytozoon bieneusi*	Dog	108	CM, PCR	33.3	–	–	(37)
	Cat	104	CM, PCR	23.1	–	–	
*Blastocystis* sp.	Dog	144	Culture, PCR	0.0	–	–	(38)
	Dog	21	Culture, PCR	0.0	–	–	(39)
	Dog	130	CM	3.1	–	–	(28)
	Cat	155	PCR	2.6		ST3 (1), ST14 (3)	(38)
	Cat	8	Culture, PCR	0.0	–	–	(39)

CM, Conventional microscopy.

a Police dog.

b Domestic dog.

## 2. Materials and methods

### 2.1. Ethical considerations

The animal study protocol used in the present survey was approved by the Research Ethics Committee of Sohag University (Egypt) on 01.12.2019.

### 2.2. Study area and sample collection

**Figure 1:**
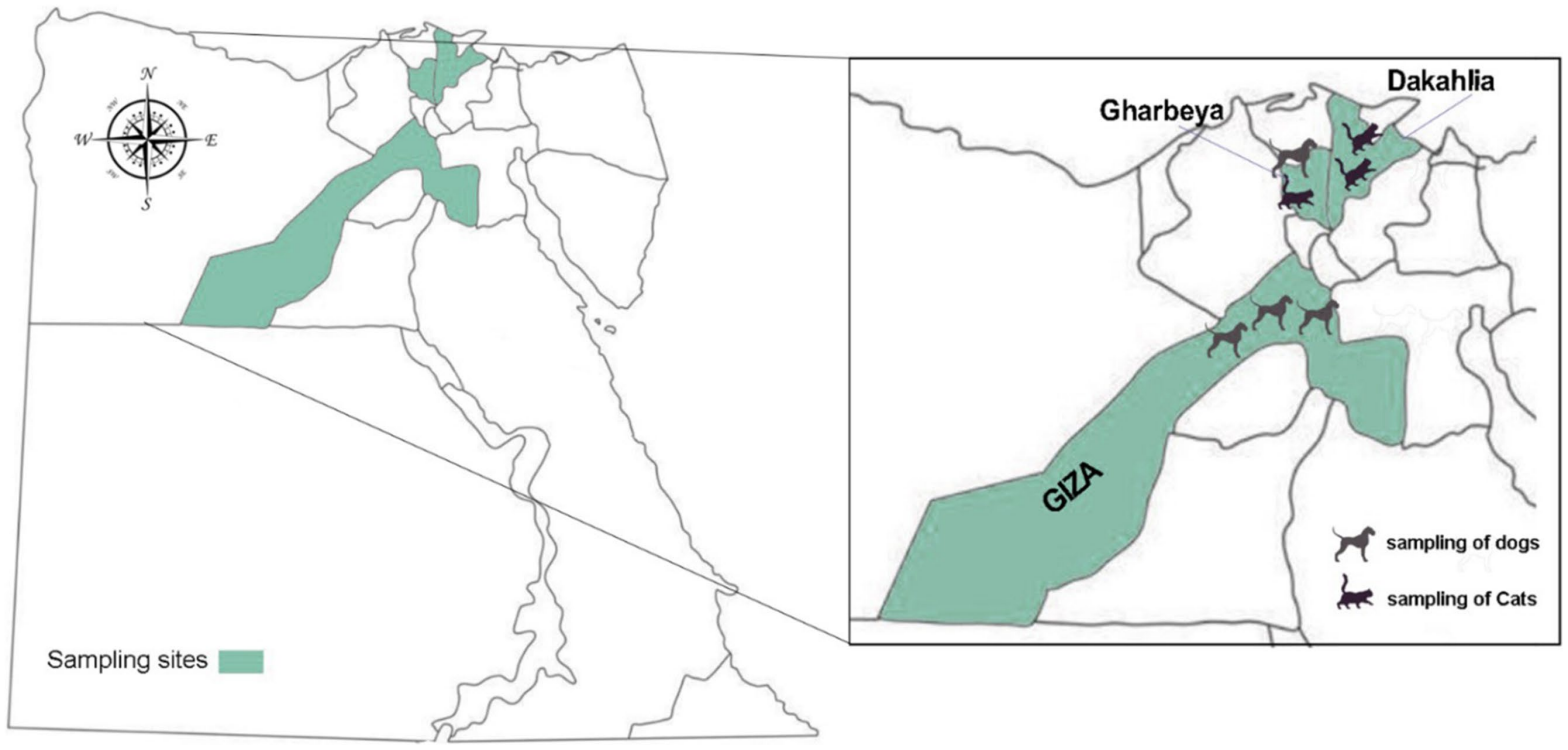
Map of Egypt showing the geographical location of the three governorates where sampling was conducted.

This is a prospective, cross-sectional study conducted during July–December 2021 in three Egyptian governorates: Dakahlia, Gharbeya, and Giza (Figure 1). A total of 352 individual fecal samples were collected from apparently healthy household dogs (*n* = 218) and cats (*n* = 134) after requesting and obtaining sampling permission from their owners. The term “household” was used to refer to those domestic animals kept in or about a dwelling house. Canine specimens were collected in Gharbeya, and Giza, whereas feline specimens were collected in Dakahlia and Gharbeya. The samples were collected freshly from the rectum of examined animals, placed into sterile plastic containers with 70% ethanol as preservative, and coded by a unique identifier. All fecal specimens included in this study were formed. Basic epidemiological data including the sex, age, and breed of the animal and the date and geographical location of sampling sites were gathered and entered into an Excel spreadsheet. Collected fecal samples were transported in refrigerated boxes to the Laboratory of Zoonoses, Faculty of Veterinary Medicine, Sohag University (Egypt) and kept at 4°C. All collected samples where then shipped to the Parasitology Reference and Research Laboratory of the National Centre for Microbiology (Majadahonda, Spain) for downstream molecular testing.

### 2.3. DNA extraction and purification

The genomic DNA was extracted from a portion (about 200 mg) of each fecal sample using the QIAamp DNA Stool Mini Kit (Qiagen, Hilden, Germany) according to the manufacturer’s guidelines, except that samples mixed with InhibitEX buffer were incubated for 10 min at 95°C. Extracted and purified DNA samples were then eluted in 200 μL of PCR-grade water and stored at 4°C until molecular analysis. The maximum time elapsed between sample collection and DNA extraction and purification was 20 weeks.

### 2.4. Molecular detection and characterization of *Cryptosporidium* spp.

Detection of *Cryptosporidium* spp. was conducted by a nested PCR protocol targeting a 587-bp fragment of the small subunit of the ribosomal RNA (*ssu* rRNA) gene of the parasite (40). Subtyping tools based on the amplification of partial sequences of the 60-kDa glycoprotein (*gp60*) gene were used to ascertain intra-species genetic diversity in samples that tested positive for *C. parvum* (41), *C. canis* (42), and *C. felis* (43) by *ssu*-PCR.

### 2.5. Molecular detection and characterization of *Giardia duodenalis*

For the identification of *Giardia duodenalis*, a real-time PCR (qPCR) protocol was used to amplify a 62-bp fragment of the *ssu* RNA gene of the parasite (44). Samples that yielded cycle threshold (C_T_) values < 32 were re-assessed using a sequence-based multilocus genotyping (MLST) scheme targeting the genes encoding for the glutamate dehydrogenase (*gdh*), β-giardin (*bg*), and triose phosphate isomerase (*tpi*) proteins to assess *G. duodenalis* molecular diversity at the sub-assemblage level. A 432-bp fragment of the *gdh* gene was amplified using a semi-nested PCR (45), while 511 and 530-bp fragments of the *bg* and *tpi* genes, respectively, were amplified with nested PCRs (46, 47).

### 2.6. Molecular detection of *Enterocytozoon bieneusi*

To identify *E. bieneusi*, a nested PCR protocol was used to amplify the ITS region as well as portions of the flanking large and small subunit of the ribosomal RNA gene as previously described (48). This procedure yielded final PCR product of 390 bp.

### 2.7. Molecular detection of *Blastocystis* sp.

*Blastocystis* sp. were detected by a direct PCR targeting a 600-bp fragment of the *ssu* rRNA gene of the parasite as described elsewhere (49).

### 2.8. PCR and gel electrophoresis standard procedures

Detailed information on the PCR cycling conditions and oligonucleotides used for the molecular identification and/or characterization of the protozoan parasites investigated in the present study is presented in Supplementary Tables 1, 2, respectively. The qPCR protocol described above was carried out on a Corbett Rotor Gene™ 6,000 real-time PCR system (QIAGEN). Reaction mixes included 2× TaqMan® Gene Expression Master Mix (Applied Biosystems, CA, United States). All the direct, semi-nested, and nested PCR protocols described above were conducted on a 2,720 Thermal Cycler (Applied Biosystems, CA, United States). Reaction mixes always included 2.5 units of MyTAQ™ DNA polymerase (Bioline GmbH, Luckenwalde, Germany), and 5–10 μL MyTAQ™ Reaction Buffer containing 5 mM dNTPs and 15 mM MgCl_2_.

### 2.9. Sequence analyses

All amplicons of the expected size were directly sequenced in both directions with the appropriate internal primer sets (see Supplementary Table 2) in 10 μL reactions using Big Dye™ chemistries and an ABI 3730xl sequencer analyzer (Applied Biosystems). Raw sequences were assembled using Chromas Lite version 2.1 software^1^ and aligned using ClustalW implemented in MEGA version 11 (50). The generated consensus sequences were compared with reference sequences deposited at the National Center for Biotechnology Information (NCBI) using the BLAST tool.^2^ Representative nucleotide sequences generated in the present study were deposited in the GenBank public repository database under accession numbers OQ778995–OQ779000 and OQ787086 (*Cryptosporidium* spp.) and OQ787087–OQ787091 (*G. duodenalis*).

### 2.10. Phylogenetic analyses

To analyze the phylogenetic relationship among various subtype families of *C. canis*, a maximum-likelihood tree was constructed using MEGA version 11 (50), based on substitution rates calculated with the general time reversible model and gamma distribution with invariant sites (G + I). Bootstrapping with 1,000 replicates was used to determine support for the clades (42).

### 2.11. Statistical analyses

The potential association between parasitic infections and the different individual risk variables (geographical location, sex, age, and breed) considered was assessed using the Fisher’s exact test. A *p*-value < 0.05 was considered as statistically significant. Analysis were conducted on the Statistical Package for the Social Sciences (SPSS) version 25 software (IBM Corporation, Armonk, NY, United States).

## 3. Results

### 3.1. Prevalence of parasites

The overall prevalences of *Cryptosporidium* spp. and *G. duodenalis* in dogs were 1.8% [4/218, 95% Confidence Interval (95% CI): 0.5–4.6] and 38.5% (84/218, 95% CI: 32.0–45.3), respectively. The overall prevalences of *Cryptosporidium* spp. and *G. duodenalis* in cats were 6.0% (8/134, 95% CI: 2.6–11.4) and 32.1% (43/134, 95% CI: 24.3–40.7). All canine and feline fecal samples analyzed tested negative for *E. bieneusi* and *Blastocystis* sp.

Dogs from Giza governorate were significantly more infected by *G. duodenalis* than their counterparts in Gharbeya governorate (*p* = 0.0006; Table 2). Cats from Dakahlia governorate were more likely to harbor infections by *G. duodenalis* than cats from Gharbeya governorate (*p* = 0.00001; Table 3). Sex, age, and breed did not affect the distribution of *Cryptosporidium* spp. in the investigated canine and feline populations. However, Persian cats were more likely to be infected by *G. duodenalis* than their counterparts from other breeds (Tables 2, 3).

**Table 2:** Distribution of *Cryptosporidium* spp. and *Giardia duodenalis* infections according to geographical origin, sex, age, and breed of examined dogs (*n* = 218).

		*Cryptosporidium* spp.			*Giardia duodenalis*		
Variable	Total (*n*)	Infected (*n*)	%	*p-*value	Infected (*n*)	%	*p-*value
**Geographical origin**							
Giza	198	4	2.0	1	83	41.9	**0.0006**
Gharbeya	20	0	0.0		1	5	
**Sex**							
Male	108	2	1.9	1	43	39.8	0.7809
Female	110	2	1.8		41	37.3	
**Age (years)**							
≤2	57	0	0.0	0.5749	24	42.1	0.5302
>5	161	4	2.5		60	37.3	
**Breed**							
Mixed	196	4	2.0	1	83	42.3	0.0527
Siberian husky	12	0	0.0		0	0.0	
German shepherd	6	0	0.0		1	16.7	
Havanese	1	0	0.0		0	0.0	
Pit bull	1	0	0.0		0	0.0	
Shih tzu	1	0	0.0		0	0.0	
Yorkshire	1	0	0.0		0	0.0	

Statistically significant values are highlighted in bold.

**Table 3:** Distribution of *Cryptosporidium* spp. and *Giardia duodenalis* infections according to geographical origin, sex, age, and breed of examined cats (*n* = 134).

		*Cryptosporidium* spp.			*Giardia duodenalis*		
Variable	Total (*n*)	Infected (*n*)	%	*p-*value	Infected (*n*)	%	*p-*value
**Geographical origin**							
Gharbeya	70	2	2.9	0.1512	6	8.6	**<0.00001**
Dakahlia	64	6	9.4		37	57.8	
**Sex**							
Male	59	2	3.4	0.4652	22	37.3	0.2692
Female	75	6	8.0		21	28.0	
**Age (months)**							
≤6	38	4	10.5	0.2224	17	44.7	0.0645
>6	96	4	4.2		26	27.1	
**Breed**							
Mixed	47	1	2.1	–	4	8.5	–
Persian	42	4	9.5	0.1842	23	54.8	**<0.00001**
Egyptian Mau	40	2	5.0	0.6761	14	35.0	0.0810
Himalayan	5	1	20.0	0.3037	2	40.0	>0.9

Statistically significant values are highlighted in bold.

Regarding co-infections, 75% (3/4) of dogs and 66.7% (4/6) of cats infected with *Cryptosporidium* spp. had concomitant infections with *G. duodenalis*.

### 3.2. Molecular characteristics of *Cryptosporidium* isolates

All four canine isolates that yielded amplicons of the expected size in *ssu*-PCR were successfully genotyped and assigned to host-specific *C. canis* by sequence analyses (Table 4). Three of them were identical to GenBank reference sequence AF112576, whereas the fourth differed from it by a single nucleotide polymorphism (SNP) at position 646. Only a single isolate could be molecularly characterized at the *gp60* locus. Sequence analysis confirmed the presence of *C. canis* subtype family XXe. The obtained nucleotide sequence differed from reference sequence MT954613 (named as XXe1) by 10 SNPs including an AGA insertion at position 226 (Table 4). We named this novel sequence as XXe2 in agreement with the established nomenclature for *Cryptosporidium* subtype families (51).

**Table 4:** Frequency and molecular diversity of *Cryptosporidium* spp. identified in the canine and feline populations investigated in the present study.

Host	Parasite species	Genotype	Subtype	No. isolates	Locus	Reference sequence	Stretch	Single nucleotide polymorphisms	GenBank ID
Dog	*C. canis*	–	–	3	*ssu* rRNA	AF112576	527–1,021	None	OQ778995
		–	–	1	*ssu* rRNA	AF112576	529–1,017	A646W	OQ778996
		XX	XXe2	1	*gp60*	MT954613	4–677	A16G, C206T, C210T, C211T, A216G, C223G, T226G, 226InsAGA, T277C, A506G	OQ787086
Cat	*C. felis*	–	–	1	*ssu* rRNA	AF108862	–	None	OQ778997
	*C. parvum*	–	–	1	*ssu* rRNA	AF112571	533–1,026	A546R, A646G, T649G, 686_689DelTAAT, T693A, A706R	OQ778998
		–	–	3	*ssu* rRNA	AF112571	573–991	A646G, T649G, 686_689DelTAAT, T693A	OQ778999
		–	–	1	*ssu* rRNA	AF112571	–	A646G, T649G, 686_689DelTAAT, T693A, C761Y	OQ779000

Del, Deletion; gp60, 60 kDa glycoprotein; Ins, Insertion; R, A/G; ssu rRNA, Small subunit ribosomal RNA; W, A/T.

**Figure 2:**
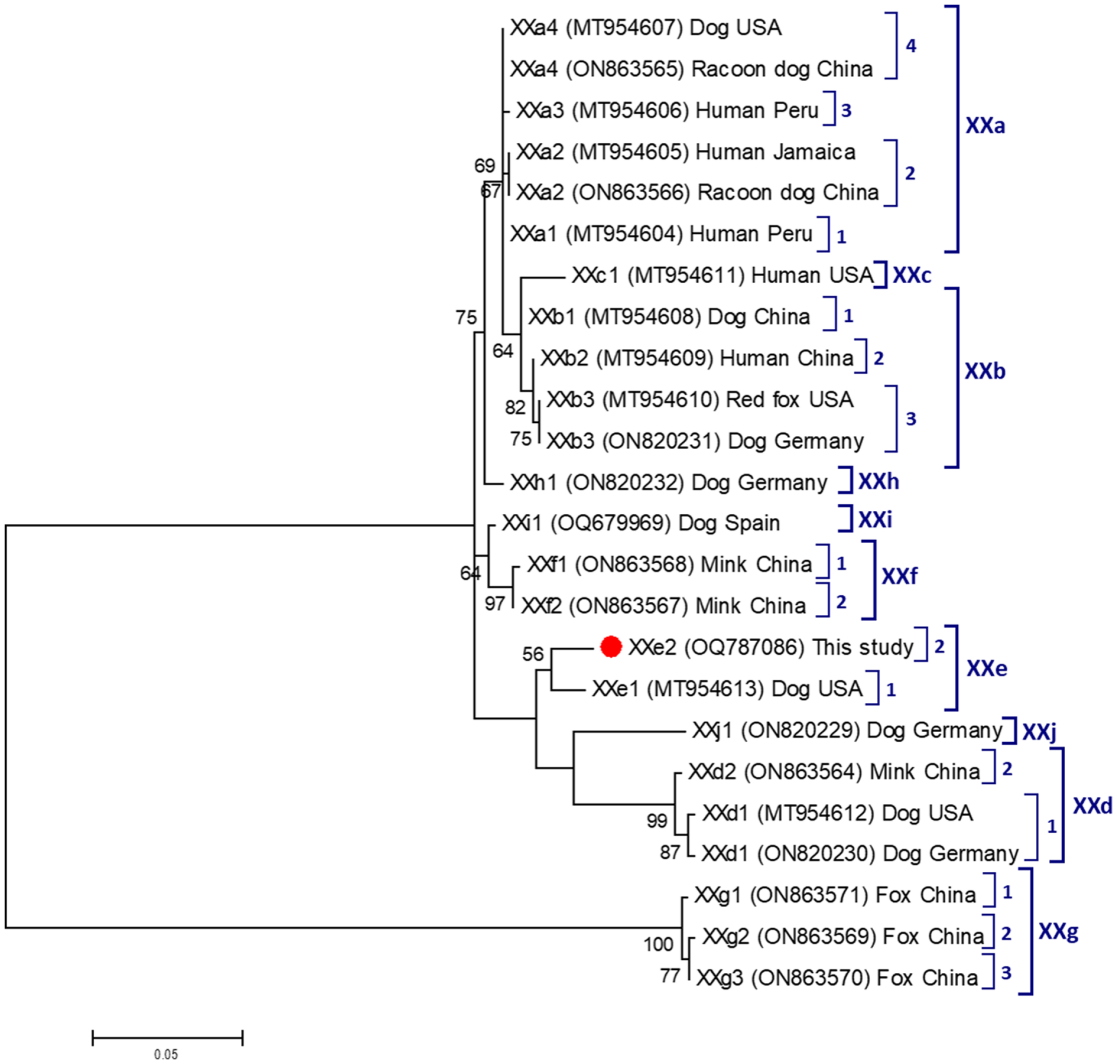
Phylogenetic relationship among nine *Cryptosporidium canis* subtype families (XXa–XXi) revealed by a maximum likelihood analysis of the partial *gp60* gene. Substitution rates were calculated by using the general time reversible model. Numbers on branches are percent bootstrapping values over 50% using 1,000 replicates. The filled red circle indicates the nucleotide sequence generated in the present study.

Figure 2 shows the maximum-likelihood tree generated with representative sequences of the nine *C. canis* subtype families (XXa, XXb, XXc, XXd, XXe, XXf, XXg, XXh, and XXi) described to date. As expected, our XXe2 isolate formed a distinct cluster with the only member (XXe1) known to belong to subtype family XXe. According to the topology of the generated tree, subtype families XXd and XXe were phylogenetically distant to the other six.

All six feline isolates that yielded amplicons of the expected size in *ssu*-PCR were successfully genotyped (Table 4). One of them was identified as *C. felis* and its nucleotide sequences showed 100% identity with reference sequence AF108862. The remaining five isolates corresponded to different genetic variants of the bovine genotype of *C. parvum* (AF112571). These five nucleotide sequences differed from AF112571 by 4–6 SNPs and all of them included the distinctive TAAT deletion at position 689 (Table 4). None of the isolates assigned to *C. felis* or *C. parvum* could be amplified at the *gp60* locus.

### 3.3. Molecular characteristics of *Giardia duodenalis* isolates

*Giardia duodenalis* qPCR-positive samples generated C_T_ values that ranged from 25.3 to 38.8 (median: 33.8; SD: 3.5) in canine samples, and from 29.2 to 40.4 (median: 36.1; SD: 2.2) in feline samples. A total of 41 fecal DNA samples with C_T_ values ≤ 32 (37 canine, 4 feline) were subjected to MLST analyses.

Among the 37 canine DNA isolates analyzed by MLST, four were successfully genotyped at the *gdh* and/or *bg* loci. Two isolates were amplified at the *gdh* locus only, one isolate was amplified at the *bg* locus only, and the remaining isolate was amplified at both loci. None of the 37 DNA isolates of canine origin could be genotyped at the *tpi* locus. Sequence analyses revealed the presence of canine-adapted assemblages C and D at equal (50%, 2/4 each) proportions (Table 5). At the *gdh* locus, the two isolates identified as assemblage C differed from reference sequence U60984 by a single SNP. The isolate assigned to assemblage D differed from reference sequence U60986 by four SNPs. Of the two isolates amplified at the *bg* locus and assigned to the assemblage D, one was identical to reference sequence AY545647, whereas the remaining one differed from it by a single SNP (Table 5). All four feline samples positive for *G. duodenalis* by qPCR failed to be amplified at the three loci (*gdh*, *bg*, and *tpi*) used for genotyping purposes.

**Table 5:** Frequency and molecular diversity of *Giardia duodenalis* identified in the canine population investigated in the present study.

Assemblage	Sub-assemblage	No. isolates	Locus	Reference sequence	Stretch	Single nucleotide polymorphisms	GenBank ID
C	–	1	*gdh*	U60984	76–491	G276A	OQ787087
	–	1	*gdh*	U60984	76–491	G282A	OQ787088
D	–	1	*gdh*	U60986	67–491	C132T, T240C, T429C, G441A	OQ787089
D	–	1	*bg*	AY545647	112–572	None	OQ787090
	–	1	*bg*	AY545647	102–590	A201G	OQ787091

*bg*, β-giardin; *gdh*, Glutamate dehydrogenase.

## 4. Discussion

Domestic dogs and cats can be a source of human infection by a wide diversity of viral, bacterial, parasitic, and fungal pathogens (52, 53). Those with unrestricted access to the outdoors might be at higher risk of pathogen exposure and represent overlooked reservoirs of zoonotic agents (54). Therefore, elucidation of the epidemiology and public health importance in these pathogens requires the use of genotyping and subtyping tools (14). Under these premises, this molecular-based study evaluated the occurrence and molecular diversity of four of the most common diarrhea-causing enteric protist parasites (*Cryptosporidium* spp., *G. duodenalis*, *E. bieneusi*, and *Blastocystis* sp.) in canine and feline populations in Egypt, with a special interest in assessing their zoonotic potential. The main strengths of the survey include (i) the use of a large sample size, (ii) the coverage of three different geographical regions, (iii) the use of highly sensitive PCRs as screening methods, and (iv) the use of specific PCR protocols for genotyping/subtyping purposes. Molecular information on the investigated protist species is particularly scarce in Egyptian animal populations in general and dogs and cats in particular. The study expands and complements information already provided by our research team on the epidemiology of enteric protists of public veterinary relevance in livestock species including buffaloes, cattle, and dromedary camels (55, 56).

*Cryptosporidium* spp. infections in Egyptian canine populations have been previously reported in the range of 2%–50% (Table 1) by conventional microscopy examination. In the only molecular-based study conducted to date, a prevalence of 24% (12/50) was found in household dogs in Sharkia Province (26). These highly variable prevalence rates are likely the reflection of changing epidemiological scenarios with differences in reservoir host populations, parasite´ strains, environmental and care conditions, sources of infection, and transmission pathways. This seems to be also the case of the present study, were *Cryptosporidium* spp. were detected at low rates (2%) in dogs from Giza governorate, but not in dogs from Gharbeya governorate. Our molecular analyses confirmed the presence of canine-adapted *C. canis* as the only *Cryptosporidium* species circulating in the surveyed dog population. This is in contrast with the evidence available in the country, where *C. parvum* was previously identified in five household dogs in Sharkia Province (26), and in two puppies with diarrhea in Qalubiya governorate (30). An asset of the present study is the use of a recently developed subtyping tool based on the amplification of partial sequences of the highly variable *gp60* gene to ascertain subtype families within *C. canis* (42). This methodology has allowed the identification of nine (XXa to XXi) subtype families of *C. canis* in a variety of animal hosts including dogs, foxes, minks, and racoon dogs, in addition to humans (42, 57, 58). The finding of *C. canis* in a number of human isolates suggests that this species might represent a public health concern for vulnerable populations such as children and immunocompromised individuals. In our study we managed to subtype one of the four *C. canis* isolates, which was assigned to novel subtype XXe2. This result contributes to expand our knowledge on the genetic diversity and host range of this *Cryptosporidium* species.

Our study represents the first PCR-based description of *Cryptosporidium* infections in domestic cats in Africa. Using molecular methods, feline cryptosporidiosis has been documented at prevalence rates of 8%–13% in the Americas including United States, of 1%–12% in Asia (mainly China), of 2%–10% in Australia, and of 5%–7% in Europe (14). The prevalence rate found in our feline population (6.0%) falls well within the range of those figures reported globally. Our molecular analyses provided interesting data. Unexpectedly, *C. parvum* was far more prevalently found than feline-adapted *C. felis* (83.3% vs. 16.7%). This is in spite of *Cryptosporidium felis* is known to be the dominant species in cats globally (14), although other *Cryptosporidium* species including *C. parvum* (59, 60), *C. muris* (61, 62), and *C. ryanae* (62) have been sporadically reported in domestic cats. Our sequence analyses revealed that all five *C. parvum* isolates corresponded to genetic variants of the bovine genotype of the parasite (63), known to have a loose host specificity and therefore a clear zoonotic potential (64). The bovine genotype of *C. parvum* accounts for 43%–100% of confirmed bovine cryptosporidiosis cases in cattle in Egypt (65–69). We hypothesize that the high proportion of feline infections by *C. parvum* detected in our feline population can be the result of cross-species transmission between cattle and domestic cats sharing habitats under high infection pressure conditions. Examples of such events have been reported in other studies (70).

Available microscopy-based epidemiological data have demonstrated the occurrence of *G. duodenalis* in 1%–32% of the canine populations investigated in Egypt (Table 1). None of these studies used PCR as screening method. In the present survey we found a higher *G. duodenalis* prevalence of 38.5%. This was an expected result, as qPCR has a superior diagnostic performance compared with microscopy (13). As in the case of *Cryptosporidium* canine infections, large differences in *G. duodenalis* prevalence rates were observed between geographical areas, with the bulk of the infections (99%) coming from the Giza governorate. Potential explanations for this finding are the higher number of samples collected in this governorate compared with those from Gharbeya, differences in animal care and wellbeing standards and even local variations in the epidemiology of the parasite including sources of infection and transmission pathways (71). Remarkably, 56% (47/84) of the canine cases of giardiasis had qPCR C_T_ values > 32, suggestive of light infections. This fact might also explain the limited number of *G. duodenalis* isolates successfully subtyped at the *gdh* and/or *bg* loci. The four *G. duodenalis* isolates characterized corresponded to canine-adapted assemblages C and D. These results are in agreement with those reporting assemblage D in four microscopy-positive household dogs visiting private pet clinics in different Egyptian governorates (35).

In the only available survey investigating the presence of *G. duodenalis* in domestic cats in Egypt, the presence of the parasite was identified by conventional microscopy in 14.8% of the animals examined (31). The global prevalence of feline giardiasis has been estimated at 2.3% (5,807/248,195) in a systematic review and meta-analysis of prevalence studies (*n* = 68) from stool samples using a variety of diagnostic methods including light microscopy, IFA, ELISA, and PCR (13). We found a much higher prevalence of 32.1% using a highly sensitive qPCR assay. Unfortunately, 90.7% (39/43) of the feline samples positive for *G. duodenalis* by this method yielded C_T_ values > 32, precluding us to determine the subtype of these isolates at the *gdh*, *bg*, and/or *tpi* loci.

In this study, *E. bieneusi* and *Blastocystis* sp. were undetected in the investigated canine and feline populations. These results are in contrast with those previously reported in Egypt. For instance, microsporidial spores were identified by microscopy examination of stained smears in 33.3 and 23.1% of canine and feline fecal samples, respectively (37). Subsequent nucleotide sequence analyses confirmed the presence of *E. bieneusi* and *E. intestinalis* in these host species. On the other side, *Blastocystis* sp. colonization/infection has been detected at low rates in domestic dogs by conventional microscopy (3.1%) and cats by PCR (2.6%) (28, 38), although other surveys failed to identify the presence of the protist using culture and PCR methods (38, 39).

Taking together, molecular subtyping data generated in the present study indicate that domestic dogs and cats are primarily infected with host-adapted species including *C. canis* and *G. duodenalis* assemblages C and D in the case of dogs and *C. felis* in the case of cats. These genetic variants are considered of limited, but by no means negligible, zoonotic potential, as all of them have been sporadically found in human cases of giardiasis and cryptosporidiosis (10, 64, 72). The exception of this general rule is the unusual high proportion of zoonotic *C. parvum* infections detected in cats, a finding that represents a public health concern and should be further investigated. It should be noted that, out of 56 molecular studies in African countries, *C. parvum* ranked second after *C. hominis* as the most prevalent *Cryptosporidium* species circulating in humans (71).

Our results showed that canine and feline populations from Gharbeya governorate harbored lower parasitic prevalence rates than their counterparts from Dakahlia and Giza governorates. These discrepancies might be attributed to differences in sample size or the sanitary conditions under which the animals were kept (71). This study has some methodological limitations that should be taken into consideration when interpreting the obtained results and the conclusions reached. First, sample size varied among sampling areas, potentially biasing the accuracy of the statistical analyses conducted. Second, sample storage and transportation conditions might have altered the quality and quantity of available parasitic DNA, compromising the performance of the molecular methods used. Finally but not least, suboptimal amount of parasitic DNA might have hampered the PCR methods used for subtyping purposes, all of them based on the amplification of single copy genes including *gdh*, *bg*, and *tpi* (for *G. duodenalis*) or *gp60* (for *Cryptosporidium* spp.).

## 5. Conclusion

This is one of the few molecular-based epidemiological surveys assessing the role of domestic dogs and cats as potential reservoirs of human infections by diarrhea-causing enteric protist parasites of public veterinary health relevance in Egypt. The main contribution of the study to the field include: (i) the confirmation that *G. duodenalis*, and to a lesser extent, *Cryptosporidium* spp. infections are common in household dogs and cats, (ii) the first description of the occurrence and molecular diversity of *Cryptosporidium* spp. infections in domestic cats in Africa, (iii) dogs are infected by canine-adapted pathogens, but cats carried an unusual high proportion of infections with zoonotic *C. parvum* that might represent a public health concern, (iv) the first description of *C. canis* subtype XXe2, and (v) the confirmation that strict carnivores such as dogs and cats are poor host species for *Blastocystis* sp. Molecular epidemiological data presented here might be useful for assenting health authorities and policy makers in designing and implementing effective intervention strategies against these zoonotic pathogens in Egypt. Simple and easy to implement measures include adequate hygiene practices (adequate canine and feline waste disposal, regular hand washing) and routine veterinary care are essential to prevent enteric parasite infections and minimize the risk of zoonotic transmission. Further research should explore the role of other domestic and wildlife species as potential reservoirs of human infections by enteric protists.

## Data availability statement

The datasets presented in this study can be found in online repositories. The names of the repository/repositories and accession number(s) can be found at: https://www.ncbi.nlm.nih.gov/nuccore; OQ778995–OQ779000, OQ787086, and OQ787087–OQ787091.

## Ethics statement

This study was approved by the Research Ethics Committee of the Faculty of Veterinary Medicine, Sohag University (Egypt) on 01.12.2019. Written informed consent was obtained from the owners for the participation of their animals in this study.

## Author contributions

EE, AGa, AA-O, AGo, SM, YM, and ME collected the samples. EE, PCK, CH-C, and BB conducted laboratory experiments. PCK, AD, and LX conducted sequence analyses. EE, AD, JA, and CH-C conducted statistical analyses. MM and EH secured the funding for conducting sampling and experimental work. EE, DG-B, and DC designed and supervised the experiments. EE and DC wrote and prepared the original draft. EE, AGa, SM, AD, DG-B, LX, and DC wrote, reviewed, and edited the manuscript. All authors contributed to the article and approved the submitted version.

## Funding

This study was partially funded by the Health Institute Carlos III (ISCIII), Spanish Ministry of Economy and Competitiveness under project PI19CIII/00029. This study was supported by Researchers Supporting Project number (RSPD2023R655), King Saud University, Riyadh, Saudi Arabia.

## Conflict of interest

The authors declare that the research was conducted in the absence of any commercial or financial relationships that could be construed as a potential conflict of interest.

## Publisher’s note

All claims expressed in this article are solely those of the authors and do not necessarily represent those of their affiliated organizations, or those of the publisher, the editors and the reviewers. Any product that may be evaluated in this article, or claim that may be made by its manufacturer, is not guaranteed or endorsed by the publisher.

## Footnotes

^1^ http://chromaslite.software.informer.com/2.1

^2^ http://blast.ncbi.nlm.nih.gov/Blast.cgi
