# Supplementary material for: Molecular characteristics and zoonotic potential of enteric protists in domestic dogs and cats in Egypt
Source: Front Vet Sci. 2023 Jul 6;10:1229151. doi: 10.3389/fvets.2023.1229151 (PMC10357006; doi:10.3389/fvets.2023.1229151)
Supplement: Supplementary file 1 [file Table_1.DOCX]

**Supplementary Table 1.** PCR cycling conditions used for the molecular identification and/or characterization of the protist species investigated in this study.

|  |  | **Temperature and time** | | | | |  |  |  |
| --- | --- | --- | --- | --- | --- | --- | --- | --- | --- |
| **Target organism** | **Locus** | | **Initial denaturation** | **Denaturation** | **Annealing** | **Extension** | **No. cycles** | **Final extension** | **Reference** |
| *Cryptosporidium* spp. | *ssu* rRNA | | 94°C 3 min | 94°C 40 s | 50°C 40 s | 72°C 1 min | 35 | 72°C 10 min | 40 |
|  | *gp60* | | 94°C 5 min | 94°C 45 s | 59/50°C 45 s | 72°C 1 min | 35 | 72°C 10 min | 41 |
|  | *gp60* | | 94°C 5 min | 94°C 45 s | 52°C 45 s | 72°C 80 s | 35 | 72°C 10 min | 42 |
|  | *gp60* | | 95°C 4 min | 95°C 30 s | 55°C 30 s | 72°C 90 s | 35 | 72°C 7 min | 43 |
| *Giardia duodenalis* | *ssu* rRNA | | 95°C 15 min | 95°C 15 s | 60°C 1 min | 72°C 30 s | 45 | – | 44 |
|  | *gdh* | | 95°C 3 min | 95°C 30 s | 55°C 30 s | 72°C 1 min | 35 | 72°C 7 min | 45 |
|  | *bg* | | 95°C 7 min | 95°C 30 s | 65/55°C 30 s | 72°C 1 min | 35 | 72°C 7 min | 46 |
|  | *tpi* | | 94°C 5 min | 94°C 45 s | 50°C 45 s | 72°C 1 min | 35 | 72°C 10 min | 47 |
| *Enterocytozoon bieneusi* | ITS | | 94ºC 3 min | 94ºC 30 s | 57/55ºC 30 s | 72ºC 40 s | 35 | 72ºC 10 min | 48 |
| *Blastocystis* sp. | *ssu* rRNA | | 95ºC 3 min | 94ºC 1 min | 59ºC 1 min | 72ºC 1 min | 30 | 72°C 2 min | 49 |

*bg*: β-giardin; *gdh*: Glutamate dehydrogenase; ITS: Internal transcribed spacer; *gp60*: 60 kDa glycoprotein; *ssu* rRNA: Small subunit ribosomal RNA; *tpi*: Triose phosphate isomerase.
