# Supplementary material for: Molecular characteristics and zoonotic potential of enteric protists in domestic dogs and cats in Egypt
Source: Front Vet Sci. 2023 Jul 6;10:1229151. doi: 10.3389/fvets.2023.1229151 (PMC10357006; doi:10.3389/fvets.2023.1229151)
Supplement: Supplementary file 2 [file Table_2.DOCX]

**Supplementary Table 2.** Oligonucleotides used for the molecular identification and/or characterization of the protist species investigated in this study.

| **Target organism** | **Locus** | **Oligonucleotide** | **Sequence (5´–3´)** | **Reference** |
| --- | --- | --- | --- | --- |
| *Cryptosporidium* spp. | *ssu* rRNA | CR-P1 | CAGGGAGGTAGTGACAAGAA | 40 |
|  |  | CR-P2 | TCAGCCTTGCGACCATACTC |  |
|  |  | CR-P3 | ATTGGAGGGCAAGTCTGGTG |  |
|  |  | CPB-DIAGR | TAAGGTGCTGAAGGAGTAAGG |  |
|  | *gp60* | AL3531 | ATAGTCTCCGCTGTATTC | 41 |
|  |  | AL3535 | GGAAGGAACGATGTATCT |  |
|  |  | AL3532 | TCCGCTGTATTCTCAGCC |  |
|  |  | AL3534 | GCAGAGGAACCAGCATC |  |
|  | *gp60* | GP60 Ccanis_F1 | ATACTCTGGTCTCCCGTTT | 42 |
|  |  | GP60 Ccanis_R1 | GTACTCGGAAGCGGTGTA |  |
|  |  | GP60 Ccanis_F2 | AAGGCGCCTCACTCATT |  |
|  |  | GP60 Ccanis_R2 | TCAGTTAGATATCACCCATTAA |  |
|  | *gp60* | GP60 CF_F1 | TTTCCGTTATTGTTGCAGTTGCA | 43 |
|  |  | GP60 CF_R1 | ATCGGAATCCCACCATCGAAC |  |
|  |  | GP60 CF_F2 | GGGCGTTCTGAAGGATGTAA |  |
|  |  | GP60 CF_R2 | CGGTGGTCTCCTCAGTCTTC |  |
| *Giardia duodenalis* | *ssu* rRNA | Probe | FAM–CCCGCGGCGGTCCCTGCTAG–BHQ1 | 44 |
|  |  | Gd-80F | GACGGCTCAGGACAACGGTT |  |
|  |  | Gd-127R | TTGCCAGCGGTGTCCG |  |
|  | *gdh* | GDHeF | TCAACGTYAAYCGYGGYTTCCGT | 45 |
|  |  | GDHiF | CAGTACACCTCYGCTCTCGG |  |
|  |  | GDHiR | GTTRTCCTTGCACATCTCC |  |
|  | *bg* | G7_F | AAGCCCGACGACCTCACCCGCAGTGC | 46 |
|  |  | G759_R | GAGGCCGCCCTGGATCTTCGAGACGAC |  |
|  |  | G99_F | GAACGAACGAGATCGAGGTCCG |  |
|  |  | G609_R | CTCGACGAGCTTCGTGTT |  |
|  | *tpi* | AL3543 | AAATIATGCCTGCTCGTCG | 47 |
|  |  | AL3546 | CAAACCTTITCCGCAAACC |  |
|  |  | AL3544 | CCCTTCATCGGIGGTAACTT |  |
|  |  | AL3545 | GTGGCCACCACICCCGTGCC |  |
| *Enterocytozoon bieneusi* | ITS | EBITS3 | GGTCATAGGGATGAAGAG | 48 |
|  |  | EBITS4 | TTCGAGTTCTTTCGCGCTC |  |
|  |  | EBITS1 | GCTCTGAATATCTATGGCT |  |
|  |  | EBITS2.4 | ATCGCCGACGGATCCAAGTG |  |
| *Blastocystis* spp. | *ssu* rRNA | BhRDr | GAGCTTTTTAACTGCAACAACG | 49 |
|  |  | RD5 | ATCTGGTTGATCCTGCCAGT |  |

*bg*: β-giardin; *gdh*: Glutamate dehydrogenase; *gp60*: 60-kDa glycoprotein; ITS: Internal transcribed spacer; *ssu* rRNA: Small subunit ribosomal RNA; *tpi*: Triose phosphate isomerase.
